# Further Evidence That MicroRNAs Can Play a Role in Hemophilia A Disease Manifestation: *F8* Gene Downregulation by miR-19b-3p and miR-186-5p

**DOI:** 10.3389/fcell.2020.00669

**Published:** 2020-07-30

**Authors:** Katarzyna I. Jankowska, Joseph McGill, Behnaz Pezeshkpoor, Johannes Oldenburg, Zuben E. Sauna, Chintamani D. Atreya

**Affiliations:** ^1^OBRR/DBCD/LCH in the Center for Biologics Evaluation and Research, U.S. Food and Drug Administration, Silver Spring, MD, United States; ^2^OTAT/DPPT/HB in the Center for Biologics Evaluation and Research, U.S. Food and Drug Administration, Silver Spring, MD, United States; ^3^Institute of Experimental Hematology and Transfusion Medicine, University Clinic Bonn, Bonn, Germany; ^4^Center for Rare Diseases Bonn (ZSEB), University Clinic Bonn, Bonn, Germany

**Keywords:** coagulation factor VIII, hemophilia A, microRNA, FVIII deficiency, RNA affinity purification, thrombosis

## Abstract

Hemophilia A (HA) is a *F8* gene mutational disorder resulting in deficiency or dysfunctional FVIII protein. However, surprisingly, in few cases, HA is manifested even without mutations in *F8*. To understand this anomaly, we recently sequenced microRNAs (miRNAs) of two patients with mild and moderate HA with no *F8* gene mutations and selected two highly expressing miRNAs, miR-374b-5p and miR-30c-5p, from the pool to explain the FVIII deficiency that could be mediated by miRNA-based *F8*/FVIII suppression. In this report, an established orthogonal *in vivo* RNA-affinity purification approach was utilized to directly identify a group of *F8*-interacting miRNAs and we tested them for *F8*/FVIII suppression. From this pool, two miRNAs, miR-19b-3p and miR-186-5p, were found to be upregulated in a severe HA patient with a mutation in the *F8* coding sequence and two HA patients without mutations in the *F8* coding sequence were selected to demonstrate their role in *F8* gene expression regulation in mammalian cells. Overall, these results provide further evidence for the hypothesis that by targeting the 3′UTR of *F8*, miRNAs can modulate FVIII protein levels. This mechanism could either be the primary cause of HA in patients who lack *F8* mutations or control the severity of the disease in patients with *F8* mutations.

## Introduction

Hemophilia A (HA) is an X chromosome-linked bleeding disorder that is associated with mutations in the Factor 8 (*F8*) gene leading to either reduced expression or production of a dysfunctional FVIII protein. However, several studies indicate that in about 1% of severe and about 3% of mild or moderate HA patients, no mutations were detected in the *F8* gene (El-Maarri et al., 2005; Johnsen et al., 2017), suggesting that there are other molecular mechanisms in addition to mutations in *F8* that regulate FVIII expression (Rosset et al., 2016; Jankowska et al., 2019).

Altered splice sites and deep intronic mutations have provided a plausible genetic mechanism for many HA patients with no mutations in the *F8* coding sequence (Castaman et al., 2011; Pezeshkpoor et al., 2013; Zimmermann et al., 2013). However, in some extremely rare cases, even intronic mutations are not detected. In a recent study, using Next Generation Sequencing (NGS), we demonstrated upregulation of eight microRNAs (miRNAs) in two such extremely rare HA patients compared to healthy controls (Jankowska et al., 2019). Using a reporter system where the *F8* 3′UTR is fused to the luciferase gene, we demonstrated that two miRNAs, miR-30c-5p and miR-374b-5p modulate *F8* gene expression in mammalian cells and, further, in cells that endogenously express FVIII protein, these two miRNAs can reduce the expression of *F8* mRNA and FVIII protein (Jankowska et al., 2019).

Numerous studies have implicated miRNAs in the regulation of eukaryotic genes and some of the studies demonstrated that miRNAs enable the fine-tuning of gene expression rather than working as on–off switches (Sevignani et al., 2006; Davis et al., 2015). Given the physiological role of FVIII, where low levels of the protein result in a bleeding disorder (Carcao, 2012; Mingot-Castellano, 2019), while high levels are associated with thrombosis (Rietveld et al., 2019), it is likely that miRNA-mediated *F8* gene fine-tuning plays a particularly important role in maintaining homeostasis. Consequently, dysregulation of miRNA levels could contribute to disease outcome(s).

A salient feature of miRNA-facilitated gene regulation is that multiple miRNAs can target the same gene and multiple genes are targeted by the same miRNA (Peter, 2010; Hashimoto et al., 2013). Consequently, it is highly unlikely that one will identify a single miRNA or a set of miRNAs whose dysregulation can explain the modulation of FVIII levels in all individuals across the globe. Given the complexity of miRNA-mediated gene regulation and the repertoire of miRNAs potentially involved, it is important to use orthogonal approaches to identify the miRNAs that regulate specific genes. One approach is to identify miRNAs bound to a given mRNA.

There are several established *in vitro* and *in vivo* methods available to identify the binding of miRNAs to mRNAs, and none of them are perfect, nor does each method capture all the interactions. Recently using an *in vitro* miTRAP method (Braun et al., 2014), miRNA:mRNA interactions relevant to the regulation of hemostatic system (Nourse et al., 2018), which is composed of multiple components and controls of blood clotting, were identified. While *in vitro* systems are clean, unlike *in vivo* methods, they do not provide the cellular milieu in which miRNA:mRNA interactions occur under physiological conditions. Therefore, in this report, we have used an established *in vivo* experimental approach that directly identifies miRNAs which are physically bound under physiological conditions to the 3′UTR of the *F8* mRNA, namely a MS2-tagged RNA affinity purification assay (Slobodin and Gerst, 2010; Yoon et al., 2012; Yoon and Gorospe, 2016). Using this approach, a pool of endogenous miRNAs associated with the 3′UTR of *F8* mRNA were identified in HEK-293T cells. Within this pool, miR-19b-3p and miR-186-5p are also among the miRNAs that were reported to be expressed in human liver cells (Hung et al., 2015; Wang et al., 2017) where FVIII is predominantly synthesized (Orlova et al., 2013). These miRNAs were also independently identified by NGS to be present at high levels in a severe HA patient and HA patient without mutations in the *F8* gene. The functional role of these two miRNAs on the *F8* gene expression is demonstrated in this report.

## Materials and Methods

### Cell Culture

HEK-293T cells were maintained in Dulbecco’s Modified Essential Medium (DMEM) with 10% fetal bovine serum (FBS), 100 U/ml penicillin, and 100 mg/ml streptomycin (Pen-Strep). Hep-G2 cells were maintained in Minimum Essential Medium (MEM) with 10% FBS. Huh-7 cell were maintained in DMEM with 10% FBS. Epstein-Barr virus-immortalized lymphoblastoid B-cells (LCLs) obtained from a healthy subject (Pandey et al., 2013) were grown in RPMI medium supplemented with 10% FBS and Pen-Strep. HeLa cells were maintained in MEM with 10% FBS and Pen-Strep. Cells were cultured in a humidified atmosphere with 5% CO_2_ at 37°C.

### Plasmid Constructs

Complementary DNA (cDNA) representing the 3′UTR of human *F8* mRNA cloned into Gaussia luciferase reporter plasmid pEZX-MT05, and a plasmid pEZX-MR04, expressing precursor miRNA along with green fluorescent protein (eGFP) reporter gene were obtained from GeneCopoeia Inc., HEK-293T and Hep-G2 cells were transfected with the above plasmids, using Lipofectamine 3000 transfection reagent (Invitrogen) according to the manufacturer’s protocol. MS2 and MS2-GST plasmids were a gift from Dr. Myriam Gorospe, NIA, NIH. The Human *F8* 3′UTR fragment was cloned into MS2 plasmid, and HEK-293T cells were transfected with the plasmids using the Lipofectamine 2000 transfection reagent (Invitrogen) according to the manufacturer’s protocol.

### MS2-Tagged RNA Affinity Purification (MS2-TRAP) and Identification of *F8* 3′UTR-Interacting miRNAs

**FIGURE 1:**
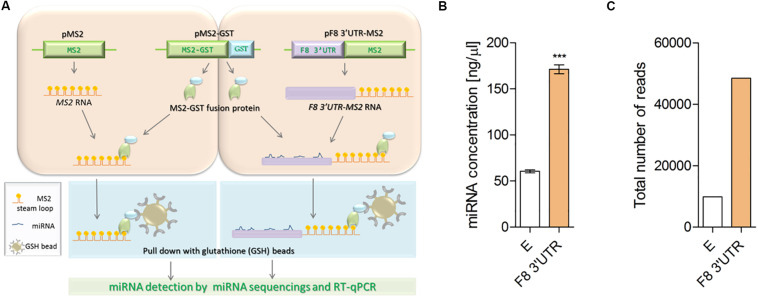
Illustration of MS2 affinity purification method. **(A)** Plasmids: pMS2 (expressing control MS2 RNA, consisting of tandem 24 MS2 hairpins, orange), pMS2-GST (expressing a fusion protein that contains MS2 RNA-recognizing portion, green), and a region (GST, glutathione S-transferase, blue) that recognizes the affinity purification reagent glutathione-SH (GSH) and pMS2-*F8* 3′UTR [expressing the test RNA of interest (purple) tagged with 24 MS2 hairpins (*F8* 3′UTR-MS2)], were transfected into HEK-293T cells for expression of the RNAs and encoded protein. HEK-293T cells were transfected with (1) the plasmids to express control RNA (MS2 RNA) and reporter protein (MS2-GST) or (2) plasmids to express the experimental RNA of interest (*F8* 3′UTR-MS2 RNA) and the reporter protein (MS2-GST). After formation of the RNA-proteins (RNP) complexes, cells were lysed and the RNPs were affinity-purified by using GSH beads. Subsequently, RNA was isolated from the complexes and subjected to next generation sequencing to identify the bound microRNAs. **(B)** Affinity purification (average of three independent experiments) demonstrated higher concentration of miRNAs in MS2-tagged *F8* 3′UTR (*E* = 60.50 ± 3.10, *N* = 4; *F8* 3′UTR = 171.30 ± 9.66, *N* = 4) and **(C)** Total read counts detected in empty control (*E* = 9886) and MS2-tagged *F8* 3′UTR (*F8* 3′UTR = 48486) of pull-down samples. ***P < 0.001.

MS2-TRAP assay was performed as described previously (Yoon et al., 2012; Yoon and Gorospe, 2016). Briefly, MS2-tagged *F8* 3′UTR (test) plasmid or empty MS2 plasmid (control), along with a MS2-GST plasmid expressing MS2 coat protein and glutathione transferase as a fusion protein, were expressed in HEK-293T cells (Figure 1). After 48 h, the harvested cells were lysed in NP-40 lysis buffer. Equal concentration of clarified supernatants (2 mg/ml of lysates) were incubated with glutathione beads overnight at 4°C to ensure binding of miRNAs to their target sequence present in the *F8* 3′UTR. Finally, the RNAs bound to the beads were extracted using Trizol reagent method as suggested by the manufacturer’s protocol (Invitrogen). RNA samples were analyzed in 2100 Agilent Bioanalyzer using the Small RNA kit (Agilent Technologies) according to the manufacturer’s instructions. All analyses were performed using the Agilent 2100 expert software. RNAs from the control and test plasmid-transfected HEK-293T cells were subjected to NGS in our in-house core facility at FDA, using Illumina small RNA kit to identify the miRNAs associated with the *F8* 3′UTR.

The NGS data was analyzed by using miRDeep2, one of the miRNA identification tools, and then we aligned this information with the miRNA database, miRBase 21. The data output was sorted based on the total number of sequence-reads for each miRNA and their fold change in the test sample relative to the control. A threshold of 30 reads in the test sample and a fourfold change between the test and control samples were used to identify miRNAs that had shown statistically significant difference between the two sets of samples.

### Luciferase Assay

HEK-293T and Hep-G2 cells were co-transfected with *F8* 3′UTR pEZX-MT05-vector and with either the precursor miRNA expression vector pEZX-MR04 containing an eGFP reporter gene or with the same vector expressing a scrambled miRNA as control, using Lipofectamine 3000. After 48 h of transfection, cell culture media were collected, and reporter gene activities were measured by a dual-luciferase assay-system (GeneCopoeia). A secreted alkaline phosphatase (SEAP) reporter expressed by a CMV promoter present in pEZX-MT05 served as the internal control to normalize for transfection efficiency. The data were presented as fold change relative to the control group. To quantify miRNA overexpression in transfected cells, quantitative PCR (qPCR) was performed as described below (Jankowska et al., 2019).

### Reverse Transcriptase Based Quantitative Real Time-PCR (RT-qPCR)

RT-qPCR was carried out as described previously (Jankowska et al., 2019). Briefly, to detect the relative levels of *F8* mRNA and miRNAs, qPCR was performed in two-step RT-PCR. First, a cDNA was generated for *F8* mRNA, using 1 μg total RNA, SuperScript III Reverse Transcriptase (Invitrogen), and oligo(dT) primers by reverse transcription.

In the second step, PCR products were quantitatively synthesized from cDNA-mRNA complexes using the TaqMan Gene Expression Master Mix and TaqMan Gene Expression Assay specific to coagulation factor VIII (Hs00240767_m1 that covers exon 1-2, Applied Biosystems) and endogenous control, GAPDH (Hs02758991_g1, Applied Biosystems), following the manufacturer’s protocol.

For mature miRNA quantification, cDNA was synthesized from total RNA samples using specific miRNA primers provided with the TaqMan MicroRNA Assay and reagents from TaqMan microRNA reverse transcription kit. The RNU6 small nuclear RNA was used as an internal control. TaqMan miRNA assays and RNU6 snRNA were from Applied Biosystems. The PCR products were amplified using the TaqMan MicroRNA Assay together with the TaqMan Universal PCR master mix. The reaction conditions were as specified by the TaqMan MicroRNA Assays Protocol. The fold change for each target gene relative to the control group was calculated using the ΔΔCt method.

### Western Blot to Detect FVIII Antigen in Lymphoblastoid B-Cells

For Western blot analysis, LCL cells that endogenously express coagulation factor VIII (Pandey et al., 2013; Jankowska et al., 2019) were lysed by 5 min pulse sonication in RIPA buffer (Cell signaling) for 20 min at 4°C, with addition of PMSF prior to lysis. Lysates were diluted in reducing sample buffer, separated on 4–12% gradient gels, transferred to nitrocellulose membranes using *Trans-*Blot Turbo system (Bio-Rad) and blocked with 5% skim milk in TPBS buffer (PBS buffer with Tween 20). Membrane-bound proteins were probed with primary antibodies specific to human coagulation factor FVIII (Abcam, ab41188, 1:1,000) overnight, followed by incubation with appropriate secondary antibodies (Goat α mouse IGg 31430 by ThermoFisher, 1:10,000). The membranes were then imaged and analyzed using an Image Station 4000MM PRO (Carestream) with Carestream MI software. Images were captured within the linear range for each probe (Jankowska et al., 2019). For loading control same blots were incubated with β-actin (Invitrogen, MA5-15739, 1:10,000), GAPDH (Abcam, ab9485, 1:10,000), and cyclophilin B conjugated with HRP (Abcam, ab205875, 1:10,000). The membranes were imaged as described above. FVIII intensities (FVIII:HC bands) were normalized to GAPDH and actin staining.

### RNA Analyses From Severe HA Patient and Healthy Volunteer Blood Samples

Blood samples from HA patients were obtained from the University Hospital (Universitätsklinikum Bonn, UKB) of Bonn, Germany. Blood samples of healthy donors were obtained from the National Institutes of Health (NIH) Blood Bank. All human blood samples were received and handled according to the protocol approved by the US-FDA’s Risk Involving Human Subjects Committee (RIHSC Protocol #16-044B). All experiments with human samples complied with: (1) The Belmont Report: Ethical Principles and Guidelines for the Protection of Human Subjects of Research, (2) the U.S. Department of Health and Human Services (HHS) regulations for the protection of human subjects at 45 CFR part 46, (3) FDA’s Federal-Wide Assurance and applicable Terms of the FWA, and (4) FDA Internal Standard Operating Procedures for FDA’s Institutional Review Board, The Research Involving Human Subjects Committee (RIHSC). An informed consent form approved by the IRB was used to obtain consent from the subject who provided blood samples.

Blood samples were collected and extracted as described previously (Jankowska et al., 2019).

The RNA samples were analyzed using Agilent 2100 expert software in 2100 Agilent Bioanalyzer using the RNA 6000 Pico and Small RNA kits, per manufacturer’s instructions (Agilent Technologies). The RNA quality was determined based on RNA Integrity Number (RIN) number. Only the samples with a RIN number of 7 and higher passed the quality control test and were used for NGS. The NGS was performed in our in-house core facility at FDA, using Illumina small RNA kit and the analyzed using miRDeep2, and then aligned with the miRNA database, miRBase 21. Aligned reads were used to establish raw read counts for each miRNA. The subjects were split into two groups, hemophiliac and healthy donors, to allow for differential miRNA expression analysis. The read counts were examined using DESeq2 (Love et al., 2014). In addition, the hemophilic group was subdivided into the group of HA patients without mutation (HAW/OM) and severe HA patient (HASEV) to distinguish miRNAs upregulated only in HA patient without mutation or in severe HA patient. A threshold of twofold change was used to discriminate differences in the expression of miRNA. A *P* < 0.05 was used to detect significant differences in the expression of miRNA as described previously (Jankowska et al., 2019). A *P* < 0.05 (non-adjusted) was used to detect significant differences in the expression of miRNA.

### Data Analysis

Statistical analysis was performed using Microsoft Excel and Prism software. Unless otherwise indicated, statistical significance was calculated using Student’s *T* test for unpaired samples, and data are presented as mean ± Std Error (Jankowska et al., 2019).

## Results

### MS2-Tagged Affinity Purification Assay Identifies *F8* 3′UTR-Bound miRNAs

The miRNAs that bind to the 3′UTR of a mRNA of interest can be identified by fusing a cDNA copy of that mRNA to one that expresses the MS2 mRNA. Co-expression of this construct with a gene expressing the MS2-GST fusion protein allows binding of the MS2 moiety to a specific stem-loop structure of the MS2-mRNA; the Glutathione S-transferases (GST) tag is used to subsequently affinity purify the complex using beads coated with Glutathione (GSH) (Figure 1A). We carried out this experiment by transfecting either MS2 alone (negative control) or MS2 fused to the 3′UTR of *F8* in HEK-293T cells. The plasmid expressing MS2-GST was also transfected in both arms. The chimeric RNAs were then affinity-purified using beads coated with GSH, mRNA concentrations were determined, and specific miRNAs bound to these mRNAs were identified by NGS (see “Materials and Methods” for details). The total miRNA concentration was found to be three-times higher in samples purified from cells transfected with the MS2-*F8* 3′UTR compared to cells transfected with MS2 alone (Figure 1B). Following NGS, the total number of miRNA-reads in the RNA sample isolated from cells transfected with the MS2-*F8* 3′UTR was 5-times higher compared to RNA isolated from the cells transfected with MS2 alone (Figure 1C).

### Identification of miRNAs Associated With MS2*-F8* 3′UTR Chimera

We have observed above that significantly larger numbers of miRNAs were obtained from cells transfected with the *F8* 3′UTR fused to MS2 compared to cells transfected with MS2 alone. We identified specific miRNAs in both groups by alignment of NGS data to the miRNA database, miRBase 21 (Table 1). The miRNAs that met both of the following criteria were considered to bind to the *F8* 3′UTR: (i) At least 30 reads for the miRNA were obtained in NGS. (ii) A Log_2_(fold change) >2 (number of reads for the miRNA from cells transfected with *F8* 3′UTR-MS2/number of reads for the miRNA from cells transfected with MS2 alone, see further details in “Materials and Methods”). Based on these criteria, we identified 64 miRNAs that can bind to the *F8* 3′UTR; these are shown in Table 1 and are ranked based on read counts in *F8* 3′UTR-MS2.

**TABLE 1:** MiRNAs identified in MS2-tagged RNA affinity assay.

miRNA	MS2-C	MS2-*F8*	Fold change	Fold change Log_2_
**hsa-miR-92a-3p**	1863	7696	4.13	2.05
hsa-miR-19b-3p	516	6122	11.86	3.57
hsa-miR-17-5p	217	4907	22.61	4.5
**hsa-miR-25-3p**	800	4244	5.31	2.41
hsa-miR-191-5p	286	3012	10.53	3.4
hsa-miR-19a-3p	282	2736	9.7	3.28
**hsa-miR-93-5p**	189	1845	9.76	3.29
hsa-miR-222-3p	154	845	5.49	2.46
**hsa-miR-7-5p**	63	673	10.68	3.42
hsa-miR-24-3p	74	662	8.95	3.16
hsa-miR-106a-5p	27	588	21.78	4.44
hsa-miR-221-3p	70	524	7.49	2.9
hsa-miR-125a-5p	97	522	5.38	2.43
**hsa-miR-196a-5p**	92	375	4.08	2.03
**hsa-miR-186-5p**	35	355	10.14	3.34
**hsa-miR-196b-5p**	46	328	7.13	2.83
**hsa-miR-130b-3p**	20	293	14.65	3.87
**hsa-miR-3607-3p**	1	278	278	8.12
hsa-miR-16-5p	10	255	25.5	4.67
hsa-let-7f-5p	55	250	4.55	2.18
hsa-miR-378a-3p	10	235	23.5	4.55
hsa-let-7g-5p	16	214	13.38	3.74
**hsa-miR-454-3p**	30	207	6.9	2.79
hsa-miR-425-5p	48	203	4.23	2.08
**hsa-miR-301a-3p**	15	182	12.13	3.6
hsa-miR-103a-3p	12	181	15.08	3.91
**hsa-miR-505-3p**	39	172	4.41	2.14
hsa-miR-18a-5p	2	167	83.5	6.38
hsa-miR-20a-5p	11	163	14.82	3.89
**hsa-miR-5701**	1	154	154	7.27
**hsa-miR-7641**	30	139	4.63	2.21
**hsa-miR-424-5p**	21	122	5.81	2.54
**hsa-miR-1275**	12	117	9.75	3.29
hsa-miR-1307-3p	19	97	5.11	2.35
hsa-miR-151a-5p	14	89	6.36	2.67
**hsa-miR-185-5p**	7	87	12.43	3.64
**hsa-miR-378a-5p**	19	85	4.47	2.16
**hsa-miR-30c-5p**	19	83	4.37	2.13
**hsa-miR-455-3p**	10	82	8.2	3.04
**hsa-miR-31-5p**	6	81	13.5	3.75
**hsa-miR-324-5p**	4	81	20.25	4.34
hsa-miR-106b-3p	11	78	7.09	2.83
hsa-miR-29a-3p	5	72	14.4	3.85
**hsa-miR-664b-3p**	1	69	69	6.11
hsa-miR-3651	2	68	34	5.09
hsa-miR-10a-5p	14	63	4.5	2.17
**hsa-miR-532-5p**	14	62	4.43	2.15
**hsa-miR-34a-5p**	7	57	8.14	3.03
hsa-miR-93-3p	10	49	4.9	2.29
**hsa-miR-1246**	10	48	4.8	2.26
hsa-miR-149-5p	7	47	6.71	2.75
hsa-miR-29c-3p	1	46	46	5.52
hsa-miR-1269b	8	45	5.63	2.49
hsa-miR-148b-3p	2	41	20.5	4.36
hsa-miR-15b-5p	2	41	20.5	4.36
**hsa-miR-361-5p**	4	38	9.5	3.25
hsa-miR-128-3p	6	37	6.17	2.62
**hsa-miR-3653-3p**	1	36	36	5.17
**hsa-miR-320a**	6	34	5.67	2.5
hsa-miR-194-5p	6	33	5.5	2.46
**hsa-miR-324-3p**	3	33	11	3.46
**hsa-miR-421**	7	32	4.57	2.19
**hsa-miR-30b-5p**	1	31	31	4.95
hsa-miR-146b-5p	5	30	6	2.58
**hsa-miR-374b-5p**	37	72	1.95	0.96
hsa-miR-144-5p	0	0		

*The list of 64 miRNAs detected in MS2-*F8* 3′UTR (MS-*F8*) sample with the highest fold change compared to control (MS2-E) with log_2_(fold change) >2 and read counts ≥30 (except miR-374b and miR-144). Bold miRNAs were predicted by prediction algorithms. The predicted miRNAs and their association scores are summarized in Supplementary Table S1. The underlined miRNAs were selected for further evaluation. Co-data for control miRNA:miR-144-5p and miR-374b-3p included for comparison.*

### Some but Not All miRNAs Associated With MS2-*F8* 3′UTR Can Be Identified Using *in silico* Tools

**FIGURE 2:**
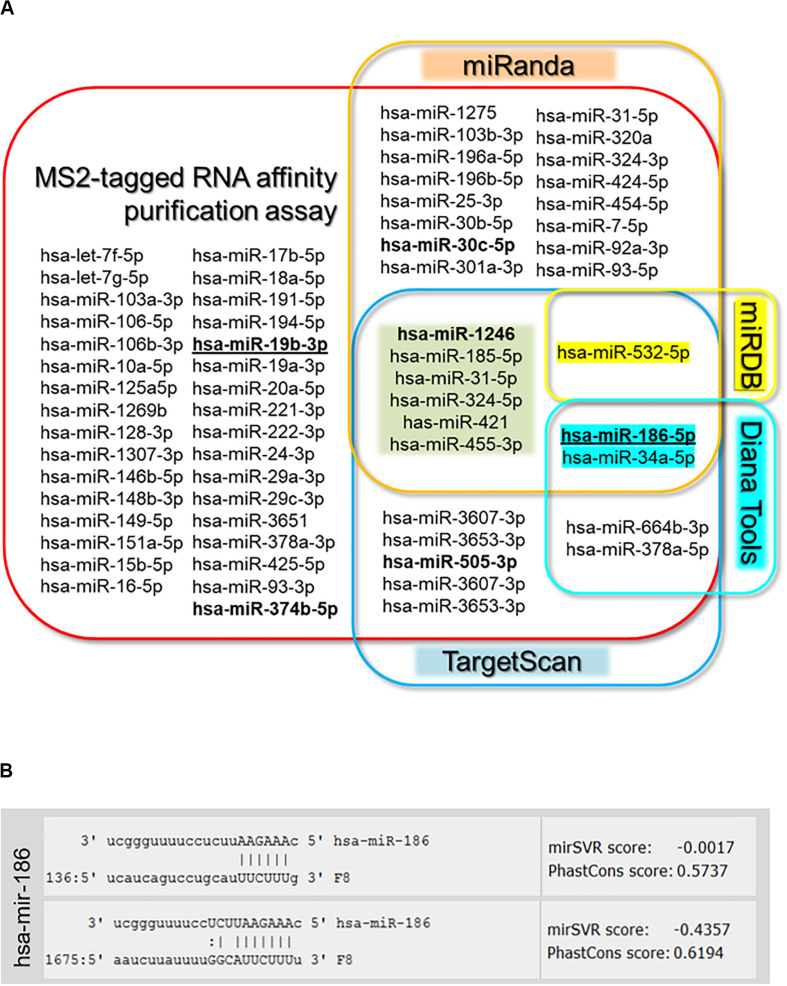
Target gene analysis. **(A)** Venn diagram of microRNAs identified by MS2-taq affinity assay and predicted to target *F8* 3′UTR by four prediction algorithms [miRanda (microrna.org), targetscan.org, Diana Tools, and miRDB]. Bold miRNAs were previously identified in HA patients (Sarachana et al., 2015; Jankowska et al., 2019), while bold underlined miRNAs were selected for further evaluation. **(B)** Sequence alignment of selected miR-186-5p and *F8*-mRNA with potential target sites in the 3′UTR of *F8* predicted by miRanda (microrna.org).

In this study, we used an orthogonal approach to identify miRNAs that can bind to the *F8* 3′UTR. Hence, we compared the results of this approach to a more commonly used *in silico* approach to identify miRNAs that can bind to the 3′UTR of specific genes. The *in silico* approaches have the advantage of speed and low cost. However, different software packages provide different results and the results are not based on physical identification of miRNA binding to the 3′UTR. We compared our results with four of the most commonly used *in silico* tools: TargetScan, miRanda, Diana Tool, and miRDB. All the miRNAs computationally determined to bind to the *F8* 3′UTR (using any of the *in silico* tools) were also identified in our experimental screen. However, additional miRNAs that were experimentally identified as binding to the *F8* 3′UTR were not predicted by the software tools. Moreover, miRNAs predicted to bind the *F8* 3′UTR using any of the software tools totaled only 50% of miRNAs identified experimentally; miRNAs that were predicted to bind the *F8* 3′UTR by at least two *in silico* methods represented only ∼14% of the miRNAs identified experimentally (Figure 2 and Supplementary Table S1). Out of 64 miRNAs identified in RNA-affinity purification approach, 6 miRNAs were predicted by both miRanda and TargetScan, and only three miRNAs were predicted by three prediction software packages. We investigated correlations (if any) between the miRNAs identified in the pull-down assay and mirSVR (miRanda) or TargetScan scores. All correlations were evaluated using ANOVA analysis of a linear regression between the two variables. The data were subset into three groups: miRNA with TargetScan scores, miRNA with mirSVR scores, and miRNA with both TargetScan and mirSVR scores. We used the read counts and the fold change (Supplementary Table S1) for correlation analyses with the TargetScan and mirSVR scores. The only significant correlation found (*P* = 0.0362) was between the log of the read counts and the TargetScan score (Supplementary Figures S1, S2).

### The miRNAs, miR-19b-3p, and miR-186-5p Associated With *F8* 3′UTR Decrease the Luciferase Signal Generated by the *F8* 3′UTR-Luciferase Reporter Plasmid

It is not practical to evaluate all the miRNAs identified as potentially binding to the *F8* 3′UTR. We selected two miRNAs for detailed characterization based on the following criteria. First, miR-19b-3p had the highest read-count among miRNAs that were identified using the experimental pull-down assay but not by *in silico* method. Second, miR-186-5p has two putative binding sites on the *F8* 3′UTR and was one of only three miRNAs to be identified in the pull-down assay as well as by three *in silico* tools, TargetScan, miRanda and Diana Tools (Figure 2). Both selected miRNAs exhibited a >10-fold read count after NGS of pulled-down miRNAs from cells transfected with the *F8* 3′UTR-MS2 compared to those transfected with MS2 alone. Finally, both these miRNAs have a likelihood of being physiologically relevant as they are both expressed in the human liver (Hung et al., 2015; Wang et al., 2017), where FVIII is predominantly synthesized (Orlova et al., 2013).

**FIGURE 3:**
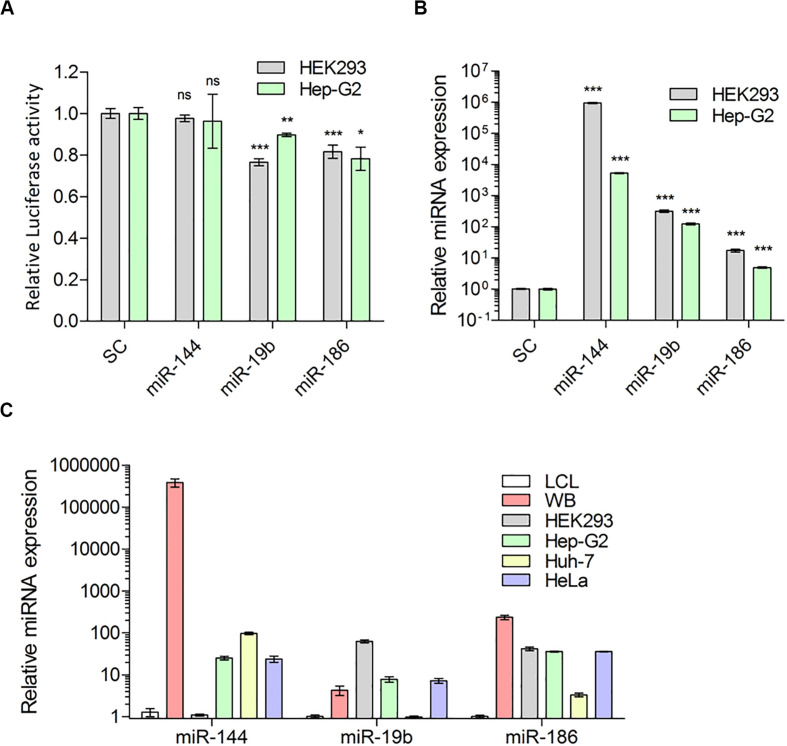
Predicted miRNAs indeed target 3′UTR of *F8*-mRNA. **(A)** Relative luciferase activity in HEK-293T and Hep-G2 cells transfected with luciferase reporter plasmid pEZX-MT05 and miRNA expression vectors (pEZX-MR04), for miR-144, miR-19b and miR-186 and scrambled miRNA as control (SC) (Mean ± SEM for HEK293: SC = 0.993 ± 0.024, *N* = 15; miR-144 = 0.977 ± 0.017, *N* = 6; miR-19b = 0.7656 ± 0.017, *N* = 6; miR-186 = 0.816 ± 0.035, *N* = 15 and for Hep-G2: SC = 0.999 ± 0.078, *N* = 6; miR-144 = 0.962 ± 0.013, *N* = 6; miR-19b = 0.897 ± 0.032, *N* = 6; miR-186 = 0.782 ± 0.055, *N* = 9). **(B)** QPCR analysis to determine overexpression level of miRNAs 72 h after transfection (Mean ± SEM for HEK-293T: SC = 1.020 ± 0.023, *N* = 4; miR-144 = 946505 ± 40246, *N* = 3; miR-19b = 320.2 ± 31.61, *N* = 3; miR-186 = 17.39 ± 1.91, *N* = 6 and for Hep-G2: SC = 1.001 ± 0.039, *N* = 4; miR-144 = 5358 ± 208.3, *N* = 3; miR-19b = 124.5 ± 8.98, *N* = 3; miR-186 = 4.392 ± 0.257, *N* = 8) and **(C)** expression of endogenous miRNAs: miR-144, miR-19b and miR-186 in whole blood (WB from two healthy donors), HEK-293T, liver cell lines: Hep-G2, Huh-7, and HeLa, relative to LCL cells (Mean ± SEM for miR-144: LCL = 1.281 ± 0.287, *N* = 6; WB = 386,400 ± 84274, *N* = 6; HEK-293 = 1.100 ± 0.057, *N* = 3; Hep-G2 = 25.39 ± 2.53, *N* = 3; Huh-7 = 97.2 ± 6.73, *N* = 3 and HeLa = 23.94 ± 4.11, *N* = 3; for miR-19b: LCL = 1.020 ± 0.089, *N* = 6; WB = 4.303 ± 1.082, *N* = 6; HEK-293 = 63.34 ± 5.204, *N* = 3; Hep-G2 = 7.820 ± 1.190, *N* = 3; Huh-7 = 0.985 ± 0.005, *N* = 3 and HeLa = 7.225 ± 0.935, *N* = 3; and for miR-186: LCL = 1.015 ± 0.075, *N* = 6; WB = 235.9 ± 28.74, *N* = 6; HEK-293 = 49.02 ± 4.074, *N* = 3; Hep-G2 = 36.14 ± 1.414, *N* = 3; Huh-7 = 3.336 ± 0.362, *N* = 3; and HeLa = 36.03 ± 0.81, *N* = 3). *P < 0.05; **P < 0.01; ***P < 0.001.

In evaluating the functional effects of miRNAs, we used miR-144-5p as a negative control because miR-144-5p is also highly expressed in human liver (Willeit et al., 2016) but does not have a target site in the *F8* 3′UTR, nor was it identified in the MS2-tagged affinity assay. The functional characterization of miR-19b-3p and miR-186-5p was carried out in two cell lines, HEK-293T and Hep-G2 (see “Materials and Methods” for details). We co-transfected cells with luciferase reporter plasmid containing the 3′UTR of human *F8* or, a control plasmid (without the *F8* 3′UTR) along with either miR-19b-3p, miR-186-5p, or miR-144-5p (negative control, see above) expression plasmids. In both cell lines, either miR-19b-3p or miR-186-5p resulted in a significant (PmiR-19b < 0.0001, PmiR-19b = 0.0060, and PmiR-186 < 0.0001, PmiR-186 = 0.0131 for HEK-293T and Hep-G2, respectively) decrease in the luciferase signal. However, co-transfection with miR-144-5p (negative control) did not significantly (*P* > 0.05, *P* = 0.435, *P* = 0.266 for HEK-293T and Hep-G2, respectively) affect the luciferase activity (Figure 3A). We confirmed the expression of all three miRNAs; miR-19b-3p, miR-186-5p, and miR-144-5p, in the transfected cells by qPCR analysis (Figure 3B). The reduction of luciferase activity was 11–23% and approximately 20% for miR-19b-3p and miR-186-5p, respectively. We also evaluated the expression level of these miRNAs in various human cell lines (Figure 3C) and observed that both miRNAs are expressed in all tested cell lines including liver Hep-G2 and Huh-7.

### The miRNAs, miR-19b-3p, and miR-186-5p Associated With *F8* 3′UTR Downregulate FVIII Expression

**FIGURE 4:**
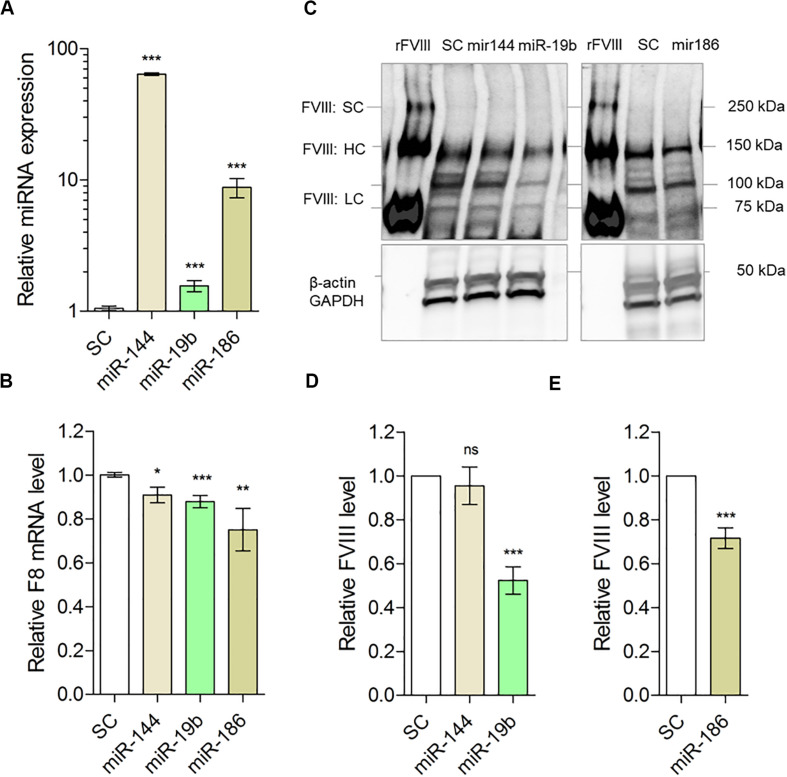
Predicted miRNAs can target *F8* and downregulate FVIII expression in mammalian cells. **(A)** Relative overexpression of miRNAs (scrambled control: SC = 1.035 ± 0.026, *N* = 14; miR-144 = 64.00 ± 1.54, *N* = 6; miR-19b = 1.562 ± 0.154, *N* = 6; miR-186 = 8.790 ± 1.468, *N* = 8) and **(B)** relative expression of *F8* mRNA (SC = 1.004 ± 0.019, *N* = 16; miR-144 = 0.9089 ± 0.0354, *N* = 12; miR-19b = 0.879 ± 0.028, *N* = 12; miR-186 = 0.751 ± 0.073, *N* = 12) in LCL cells transfected with miRNAs expression vectors compared to control cells (scrambled control, SC). Q-PCR results of miRNAs and *F8* were normalized to RNU6 and GAPDH, respectively. **(C)** Relative FVIII level in LCL cells collected 72 h after transfection with miRNAs: miR-144, miR-19b, and miR-186 expression vectors compared to control cells (SC) determined by Western blot from transfected samples along with recombinant FVIII (rFVIII). Multiple species were detected: Single chain FVIII (FVIII:SC) at about 267 kDa, heavy chain polypeptides (FVIII:HC) generated after FVIII single chain cleavage within the B domain with apparent MW range between 90 and 200 kDa; light chain (FVIII:LC) with apparent MW of 80 kDa and loading controls; and β-actin and GAPDH detected at approximately 42 and 35 kDa, respectively. The blots were cropped to improve clarity of the results. Full-length blots are presented in Supplementary Figure S3. **(D)** Quantification of FVIII heavy chain in cells transfected with miR-144 and miR-19b (SC = 1.011 ± 0.0127, *N* = 8; miR144 = 0.955 ± 0.085, *N* = 8; miR-19b = 0.523 ± 0.0623, *N* = 8) from **(C)**. **(E)** Quantification of FVIII heavy chain in cells transfected with miR-186 (SC = 0.989 ± 0.0472, *N* = 8; miR186 = 0.717 ± 0.002, *N* = 8) from **(C)**. Bands normalized to β-actin and GAPDH. *P < 0.05; **P < 0.01; ***P < 0.001.

Once the effect of downregulation by miR-186-5p and miR-19b-3p on *F8* 3′UTR-tagged luciferase expression was established, we wanted to determine the following: (i) whether ectopic expression of tested miRNAs suppresses the *F8* mRNA levels in the cells and (ii) whether the miRNAs exert their effect on the translational ability of *F8* mRNA. We used lymphoblastoid B-cells (LCL line) that endogenously express FVIII protein to address these questions. The cells were transfected with scrambled miRNA (scrambled control, SC), miR-144-5p (as negative control) and miR-19b-3p or, miR-186-5p and subsequently *F8* mRNA levels were estimated by qPCR (see “Materials and Methods”). We obtained expression of all three miRNAs; although miR144-5p and miR-186-5p expressed at higher levels than miR-19b-3p (Figure 4A), expression of miR19b-3p and miR-186-5p significantly decreased *F8* mRNA levels (miR-19b, *P* = 0.002 and miR-186, *P* = 0.008) (Figure 4B). Concomitantly, the FVIII protein levels, estimated by immunoblot analysis, were also lowered by approximately 30% and 40%, respectively, following transfection of miR-186-5p and miR-19b-3p in LCL cells (Figures 4C–E). As expected, overexpressing the negative control, miR-144-5p, in LCL cells did not result in a significant (*P* = 0.5231) change in FVIII expression compared to cells transfected with scrambled control miRNA. These results demonstrate that expression of miR-186-5p and miR-19b-3p in mammalian cells modulates FVIII protein expression.

### The miRNAs That Target the *F8* Gene and Regulate FVIII Expression Could Contribute to HA Severity in Some Patients

Further, we determined whether the 64 miRNAs we identified as being associated with the *F8* mRNA, are also expressed in HA patients relative to healthy controls. We performed miRNA sequencing analysis of blood samples from a patient with severe HA and two mild and moderate HA patients with no mutations in the *F8* gene that were previously evaluated for miRNA dysregulations (Jankowska et al., 2019). Of the 64 miRNAs identified as bound to the *F8* 3′UTR (Figure 2A), 22 miRNAs were also present at relatively high levels (>2-fold change) and eight miRNAs were present at low levels (<0.5-fold change) in the severe HA patient (Table 2) including miRNA-19b-3p and miR-186-5p. Moreover, two miRNAs were also upregulated in the HA patient without mutations in the *F8* gene; miRNA-19b-3p and miR-186-5p were elevated (with fold change >1.6). Moreover, the results described here suggest that miRNAs that target *F8* mRNA may fine-tune gene expression and may affect HA severity in some patients as degree of dysregulation of some miRNAs (Table 2) is proportional to the severity of HA. For example, mir-30c-5p, miR-93-3p, miR-1246, and others (Table 2) that were upregulated in HA patients showed increased miRNA expression with increased HA severity (Table 2). On the other hand, miR-93-5p, miR-196b-5p, let-7g-5p, miR-320a, and miR-221-3p that were downregulated in HA patients displayed decreased miRNA expression with increased HA severity. Overall, upregulation of these miRNAs in the HA patient validates the results from the MS2-tagged affinity purification assay and further confirms the role of these miRNAs in the regulation of FVIII expression.

**TABLE 2:** MiRNAs expression level (normalized read count) in HA patients compared to healthy donors.

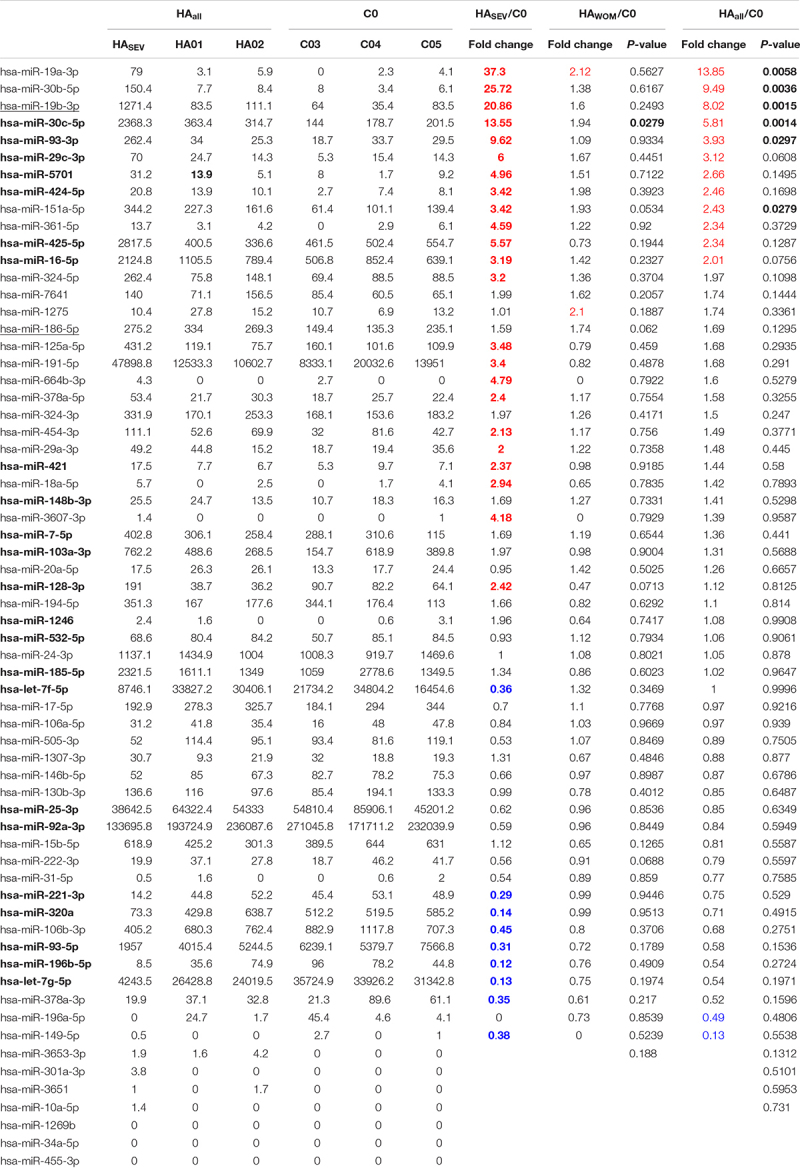

*NGS data from severe HA patient (HA_*SEV*_) and HA patient without mutation (HA_*WOM*_) in *F8* gene (HA01 = moderate HA patient; HA02 = mild HA patient) compared to healthy donors (C0, C03–C05) isolated from blood samples. (Fold change >2 is marked in red, fold change <0.5 is marked in blue. MiRNAs highlighted in bold display upregulation in miRNA expression proportional to HA severity, *P* < 0.05 are marked by bold font). The underlined miRNAs were selected for further evaluation.*

## Discussion

We have investigated the miRNA mediated control of FVIII gene expression, which involves miRNA:mRNA interactions leading to the dysregulation of mRNA functions. This interaction has important physiological implications, since both high and low levels of FVIII in an individual have clinical consequences. High levels of FVIII causes thrombosis (Kamphuisen et al., 2001; Atalay et al., 2005; Jenkins et al., 2012); persistent high levels of FVIII suggest the aberrant regulation of *F8* expression. One possible mechanism for high levels of FVIII is that those miRNAs that normally target and downregulate the *F8* gene are either expressed at low levels or not expressed at all. At the other end of the spectrum, miRNAs that target and downregulate *F8*, if overexpressed, could result in lower expression of the FVIII protein leading to the HA phonotype (Graw et al., 2005; Oldenburg et al., 2014; Nienhuis et al., 2017; Benson et al., 2018).

Several methods can be used to identify and characterize miRNA mediated control of gene expression (Calin et al., 2004; Krek et al., 2005; Grimson et al., 2007; Goff et al., 2009; Guerau-de-Arellano et al., 2012; Yoon et al., 2012; Ahmadi et al., 2013; Zheng et al., 2013; Agarwal et al., 2015; Dusl et al., 2015; Lagana, 2015; He et al., 2018). Expression levels of miRNAs can be estimated by deep-sequencing of samples from individuals diagnosed with a disease and healthy volunteers followed by statistical analyses (Gombar et al., 2012; Gyvyte et al., 2017; Jin et al., 2017; Chen et al., 2018). Such an analysis does not provide direct mechanistic evidence for miRNA-gene association. Alternatively, computational prediction software can be used to search for target miRNAs (Krek et al., 2005; Grimson et al., 2007; Maziere and Enright, 2007; Zheng et al., 2013; Lagana, 2015) to the 3′UTR of a specific gene. Although computational predictions narrow down the list of potential candidates, ultimately the miRNAs identified must be experimentally validated. In general, miRNAs engage in fine-tuning gene expression and are rarely “on” or “off” switches. Also, several miRNAs target the same UTR and several UTRs are targeted by the same miRNA (Peter, 2010; Hashimoto et al., 2013). These redundancies possibly provide the cell with a robust toolkit for maintaining homeostasis and gene fine-tuning. However, it also means that miRNA-mediated control of gene expression can be different for different individuals and vary based on their physiological state. Thus, to understand the mechanistic underpinnings of the role of miRNAs in controlling *F8* gene expression, it is important to have experimental techniques that allow the direct detection of miRNAs engaged with the *F8* UTR. Such a technique can be deployed for individual patients, cell-types and be used to interrogate the organism at different time points.

We compared the results of the MS2-tagged RNA affinity purification assay with other *in silico* methods used to identify miRNAs that bind to the *F8* 3′UTR. Out of 64 identified in the affinity purification assay, 32 miRNAs are predicted to bind the *F8* 3′UTR using four commonly used *in silico* algorithms (Table 1 and Supplementary Table S1). Recently, using NGS, we identified miRNAs predicted to bind the *F8* 3′UTR that are upregulated in HA patients with no mutations in the *F8* gene (Jankowska et al., 2019).

Taken together, our results demonstrate that the MS2-tagged RNA affinity purification assay has an added value in understanding miRNA:*F8* 3′UTR interactions (Figure 1A) by identifying the miRNAs that directly interact with mRNAs. We demonstrated that both miR-186-5p and miR-19b-3p can downregulate the *F8* gene. In addition, miR-19b-3p was also shown to be expressed at significantly (*P* < 0.05) higher levels in a severe HA patient and found to be elevated in HA patients with no mutations in *F8* gene compared to normal controls in our previous study as well (Sarachana et al., 2015). It is gratifying to note that miR-1246 which was previously shown to downregulate FVIII (Sarachana et al., 2015) was also one of the miRNAs identified using the MS2-tagged RNA affinity purification assay. Furthermore, miR-30c-5p that was previously reported to be dysregulated in HA patients without mutations and shown to regulate endogenous FVIII expression in LCL and HUVEC cell lines (Jankowska et al., 2019) was also identified in MS2-tagged RNA affinity purification assay. It needs to be mentioned here that miR-374b-5p that was previously identified in an HA patient without mutations in the *F8* was also pulled down from HEK-293 cells transfected with MS2-*F8* 3′UTR but did not meet our cut-off criteria (Table 1). Interestingly, miR-374b-5p was previously pulled-down by *F8*-3′UTR cloned into miRNA-Trap system in Huh-7 (fold change Log_2_ = 2.4) (Nourse et al., 2018). Our previous studies have shown that while this miRNA is expressed in HEK-293, the endogenous expression of this miRNA is lower than miR-30c-5p. While miR-374-5p was highly expressed in HA patients, normalized expression level of miR-374b-5p in blood samples from health donors was over 30 times lower than that observed for miR-186-5p and miR-30c-5p, and over 10 times lower than for miR-19b-3p.

Additionally, we demonstrated that the miRNAs functionally evaluated in this study are also expressed in other human cell lines including liver Huh-7 and Hep-G2. Importantly, it has been reported that these miRNAs are expressed in the human liver (Hung et al., 2015; Wang et al., 2017), where FVIII is predominantly synthesized (Orlova et al., 2013). Since these two miRNAs were able to dysregulate endogenous expression of FVIII in LCL cells and target *F8* mRNA we can reasonably postulate that these miRNAs may regulate FVIII levels in other cells including liver sinusoidal endothelial cells.

Based on predicted binding to the 3′UTRs, the two miRNAs studied here (miR-186-5p and miR-19b-3p) may also regulate expression of other proteins involved in the coagulation cascade (Supplementary Figure S4). This observation warrants further investigations; but, it does raise the possibility that the cause of bleeding disorders in some individuals may be more complex than a mutation in a single protein in the coagulation cascade. This leads to some interesting possibilities. For instance, reports have demonstrated that HA patients with the identical mutation manifest mild, moderate, or severe forms of the disease (Johnsen et al., 2017). These observed differences in the serum FVIII activity are perhaps due to differences in the levels of FVIII, plausibly regulated by miRNAs. There are also reports that *F8* genes with missense mutations that cause HA when expressed *in vitro* in heterologous expression systems do not show any differences in specific activity compared to wild-type *F8* (Ogata et al., 2011). Thus, the influence of miRNA on modulating FVIII levels and disease severity may be quite common. If this postulate is true, there are ramifications in the use of gene therapy to treat HA. It is likely that individual differences in levels of serum FVIII in gene therapy participants may be linked to their endogenous miRNA profiles.

Although our results are interesting and have implications in HA diagnosis, prognosis and care, they require additional confirmation. In several studies, we (Sarachana et al., 2015; Jankowska et al., 2019) and others (Rosset et al., 2016; Nourse et al., 2018; Jankowska et al., 2020) have shown miRNA-mediated control of *F8* gene expression. However, the clinical implications of these findings require additional studies with larger cohorts. Nonetheless, clinical correlates will be difficult to establish as a rare subpopulation of a rare disease is involved. Establishing proof-of-principle is also difficult as several miRNAs are involved in the regulation of *F8* gene expression and the repertoire of miRNAs modulating FVIII levels may not be the same in all individuals.

Taken together, our current and previous studies (Sarachana et al., 2015; Jankowska et al., 2019) highlight the role of miRNAs in FVIII expression and provide a plausible explanation that besides the coding sequence of *F8*, non-coding regions and other regulatory elements should also be studied to fully understand the causes and consequences of dysregulation of *F8* gene expression. NGS studies of blood sample confirmed dysregulation of these miRNAs in an HA patient. Interestingly, the level of dysregulation of some miRNAs in HA patients were proportional to the degree of HA severity, which suggest that upregulation of some miRNA may intensify the clinical phenotype of HA. The fact that FVIII expression can be controlled by miRNAs is important not only for HA patients, but also for patients with thrombosis in which the FVIII level is elevated. Targeting these RNAs could present new therapeutic possibilities. Additional quantitative studies on a larger group of HA patients with specific mutations and specific degree of severity as well as on patients with thrombosis will be needed to fully discover the pool of miRNAs that target *F8* mRNAs and affect the protein expression leading to bleeding disorders or thrombosis.

## Data Availability Statement

The original contributions presented in the study are included in the article/Supplementary Material, further inquiries can be directed to the corresponding author/s.

## Ethics Statement

All human blood samples were received and handled according to the protocol approved by the US-FDA’s Risk Involving Human Subjects Committee (RIHSC Protocol #16-044B). All experiments with human samples complied with: (1) The Belmont Report: Ethical Principles and Guidelines for the Protection of Human Subjects of Research, (2) the U.S. Department of Health and Human Services (HHS) regulations for the protection of human subjects at 45 CFR part 46, (3) FDA’s Federal-wide Assurance and applicable Terms of the FWA, and (4) FDA Internal Standard Operating Procedures for FDA’s Institutional Review Board, The Research Involving Human Subjects Committee (RIHSC). An informed consent form approved by the IRB was used to obtain consent from the subject who provided blood samples. The patients/participants provided their written informed consent to participate in this study.

## Author Contributions

KJ helped in the design of the study, performed data analyses, and participated in writing of the manuscript. JM participated in the bioinformatics/statistical analyses of sequenced samples. BP and JO carried out the clinical studies including recruitment of HA patients, preparation of clinical samples, and assisted in the review and editing of the manuscript. ZS and CA designed the research plan, oversaw the project, and wrote the manuscript. All authors contributed to the article and approved the submitted version.

## Disclaimer

The findings and conclusions in this article have not been formally disseminated by the Food and Drug Administration and should not be construed as representing any Agency determination or policy.

## Conflict of Interest

The authors declare that the research was conducted in the absence of any commercial or financial relationships that could be construed as a potential conflict of interest.
