# Supplementary material for: Further Evidence That MicroRNAs Can Play a Role in Hemophilia A Disease Manifestation: F8 Gene Downregulation by miR-19b-3p and miR-186-5p
Source: Front Cell Dev Biol. 2020 Jul 30;8:669. doi: 10.3389/fcell.2020.00669 (PMC7406646; doi:10.3389/fcell.2020.00669)
Supplement: Supplementary file 1 [file Data_Sheet_1.docx]

Supplementary Material

**MicroRNA mediated gene fine-tuning: A case study with Hemophilia A causing *F8* gene regulation by miR-19b and miR-186**

# Katarzyna I Jankowska1, Joseph McGill2, Behnaz Pezeshkpoor3,4, Johannes Oldenburg3,4, Zuben E. Sauna2 and Chintamani D. Atreya1*

1OBRR/DBCD/LCH and 2OTAT/DPPT/HB in the Center for Biologics Evaluation and Research, US Food and Drug Administration, Silver Spring, MD, United States of America,

3Institute of Experimental Hematology and Transfusion Medicine, University Clinic Bonn, Bonn Germany

4Center for Rare Diseases Bonn (ZSEB), University Clinic Bonn, Bonn, Germany

*** Correspondence:**Chintamani D. Atreya
[Chintamani.Atreya@fda.hhs.gov](mailto:corresponding.author@email.example)


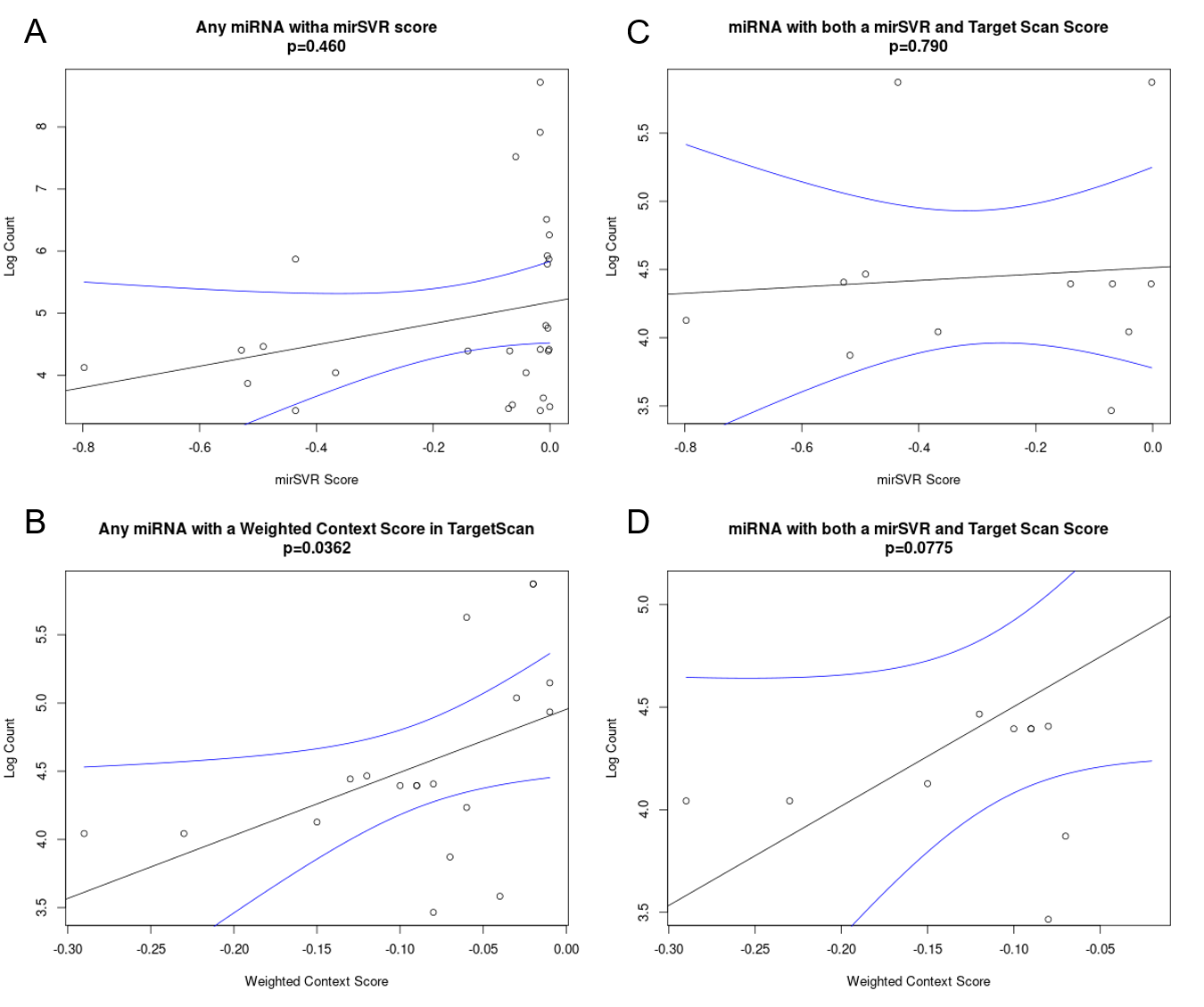


**Supplemental Figure S1. Correlations analysis between raw read counts and miRanda (mirSVR) or TargetScan scores.**

The selected miRNAs predicted to target *F8* mRNA (Table 1) were investigated for correlations between raw read counts vs control, and miRanda (mirSVR) or TargetScan scores (Weighted context++ score). All correlations were evaluated using ANOVA analysis of a linear regression between the two variables. The data were subset into three groups: miRNA with mirSVR scores (A), miRNA with TargetScan scores (B) and miRNA with both TargetScan and mirSVR scores (C, D). Additionally, in cases where miRNA had two score for mirSVR, the lower score was kept under the assumption that it would be the more likely binder. Of all of these analyses, the only significant correlation found(p=0.0362) was between the log of the read counts and the TargetScan score. This correlation was less obvious when you subset the data to only include miRNA which had both TargetScan and mirSVR scores. This lessening in confidence of a correlation between the factors could be due to a decrease in the amount of data available for the comparison.


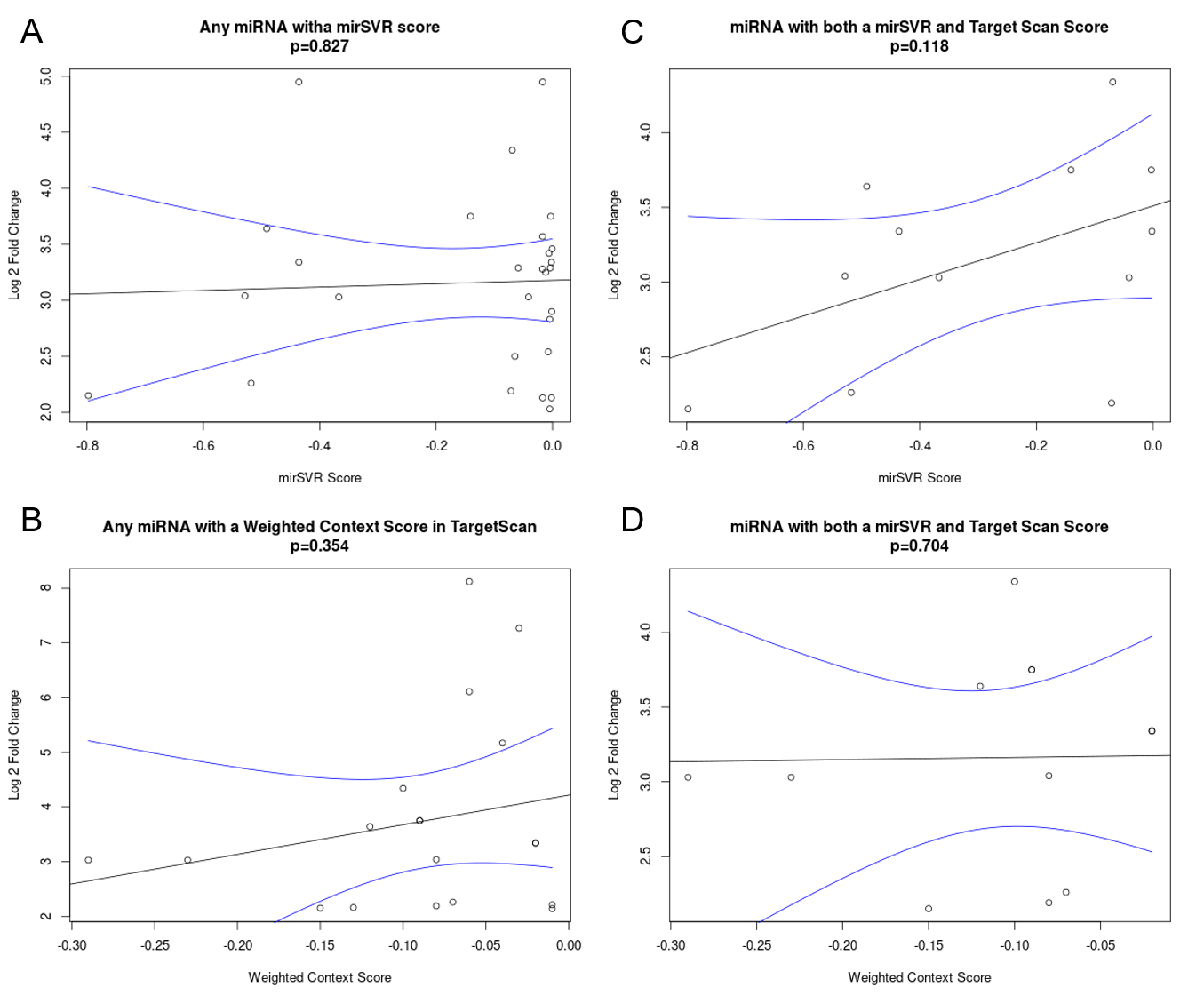


**Supplemental Figure S2. Correlations analysis between fold change and miRanda (mirSVR) or TargetScan scores.**

The selected miRNAs predicted to target *F8* mRNA (Table 1) were investigated for correlations between fold change vs control, and miRanda (mirSVR) or TargetScan scores (Weighted context++ score). All correlations were evaluated using ANOVA analysis of a linear regression between the two variables. The data were subset into three groups: miRNA with mirSVR scores (A), miRNA with TargetScan scores (B) and miRNA with both TargetScan and mirSVR scores (C, D). Additionally, in cases where miRNA had two score for mirSVR, the lower score was kept under the assumption that it would be the more likely binder.

|  |  | **miRanda** | **TargetScan** | **Diana Tools** | **miRDB** |
| --- | --- | --- | --- | --- | --- |
| miRNA | Fold change Log2 | mirSVR score: | Weighted context++ score | miTG score | Target score |
| hsa-miR-92a-3p | 2.05 | -0.0063 |  |  |  |
| hsa-miR-25-3p | 2.41 | -0.0076 |  |  |  |
| hsa-miR-93-5p | 3.29 | -0.0586 |  |  |  |
| hsa-miR-7-5p | 3.42 | -0.0058 |  |  |  |
| hsa-miR-196a-5p | 2.03 | -0.0045 |  |  |  |
| hsa-miR-186-5p | 3.34 | -0.4357 | -0.02 | 0.7384 |  |
|  |  | -0.0017 |  |  |  |
| hsa-miR-196b-5p | 2.83 | -0.0045 |  |  |  |
| hsa-miR-130b-3p | 3.87 | -0.0526 |  |  |  |
| hsa-miR-3607-3p | 8.12 |  | -0.06 |  |  |
| hsa-miR-454-3p | 2.79 | -0.0537 |  |  |  |
| hsa-miR-301a-3p | 3.6 | -0.052 |  |  |  |
| hsa-miR-505-3p | 2.14 |  | -0.01 |  |  |
| hsa-miR-5701 | 7.27 |  | -0.03 |  |  |
| hsa-miR-7641 | 2.21 |  | -0.01 |  |  |
| hsa-miR-424-5p | 2.54 | -0.0071 |  |  |  |
| hsa-miR-1275 | 3.29 | -0.0036 |  |  |  |
| hsa-miR-185-5p | 3.64 | -0.491 | -0.12 |  |  |
| hsa-miR-378a-5p | 2.16 |  | -0.13 | 0.7017 |  |
| hsa-miR-30c-5p | 2.13 | -0.0166 |  |  |  |
|  |  | -0.0016 |  |  |  |
| hsa-miR-455-3p | 3.04 | -0.5284 | -0.08 |  |  |
| hsa-miR-31-5p | 3.75 | -0.1406 | -0.09 |  |  |
|  | 3.75 | -0.0028 |  |  |  |
| hsa-miR-324-5p | 4.34 | -0.0689 | -0.1 |  |  |
| hsa-miR-664b-3p | 6.11 |  | -0.06 | 0.7415 |  |
| hsa-miR-532-5p | 2.15 | -0.7977 | -0.15 |  | 65 |
| hsa-miR-34a-5p | 3.03 | -0.3671 | -0.29 | 0.8185 |  |
|  |  | -0.041 | -0.23 |  |  |
| hsa-miR-1246 | 2.26 | -0.5177 | -0.07 |  |  |
| hsa-miR-361-5p | 3.25 | -0.0114 |  |  |  |
| hsa-miR-3653-3p | 5.17 |  | -0.04 |  |  |
| hsa-miR-320a | 2.5 | -0.0644 |  |  |  |
| hsa-miR-324-3p | 3.46 | -0.0003 |  |  |  |
| hsa-miR-421 | 2.19 | -0.071 | -0.08 |  |  |
| hsa-miR-30b-5p | 4.95 | -0.0166 |  |  |  |
|  |  | -0.4357 |  |  |  |

**Table S1.** **MiRNAs (32) predicted by at least one perdition software.** Among this group, 5 miRNAs were predicted to target 3’UTR of *F8* only by TargetScan, 16 miRNAs only by miRanda (microRNA.org), and 6 by both. 2 miRNAs were predicted by Diana Tools and TargetScan, 2 miRNAs predicted by Diana Tools, Target Scan and miRanda. Only one miRNA was predicted by miRBD, TargetScan and miRanda (as shown in Figure 2). The miRNAs-*F8* association is scored by mirSVR score in miRanda (This score is an estimate of the miRNA effect on the mRNA expression level. The more negative the score, the greater effect) and by Weighted context++ score for Target Scan (The scores with a lower negative value indicate a greater prediction of repression). MiTG score for Diana Tools and TargetScore for miRDB.

**
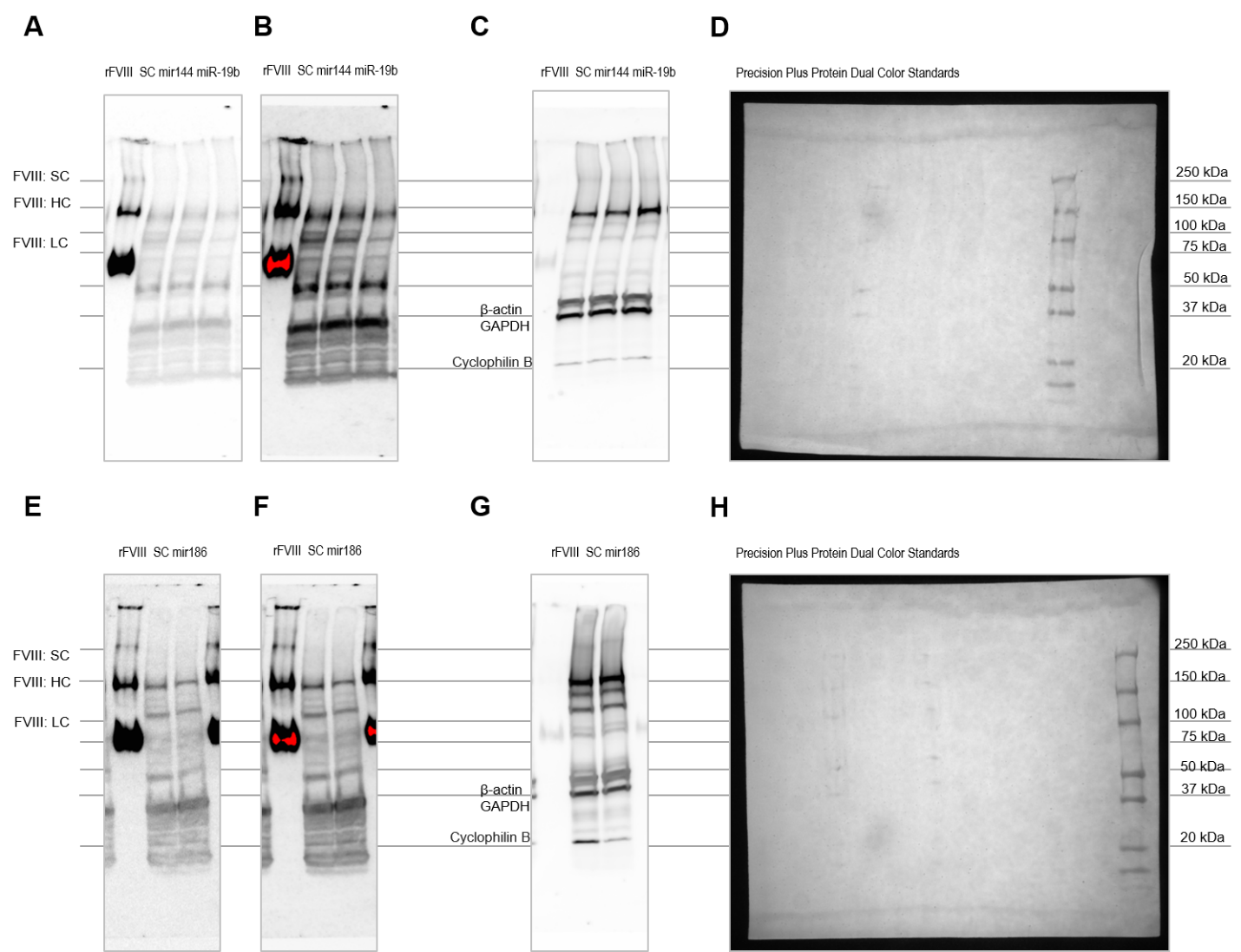
**

**Supplemental Figure S3. Full-length blots from Figure 4C**

(A-B and E-F) Western Blot of FVIII expression in LCL cells collected 72h after transfection with miRNAs: miR-144, miR-19b (A-D) and miR-186 (E-H) compared to control cells (SC) and recombinant FVIII (rFVIII) after 1min (A, E) or 10 min (B, F). exposure. Multiple species were detected: Single chain FVIII (FVIII:SC) at about 267kDa, heavy chain polypeptides (FVIII:HC) generated after FVIII single chain cleavage within the B domain with apparent MW range between 90-200 kDa, light chain (FVIII:LC) with apparent MW of 80kDa (C, G) Loading controls: Same blots after incubation with β-actin and GAPDH and cyclophilin B antibodies. (D, H) Precision Plus Protein Dual Color Standards (BioRad). Analyzed bands for loading controls: β-actin, GAPDH and cyclophilin B detected at approximately 42, 35 and 21kDa, respectively.

**
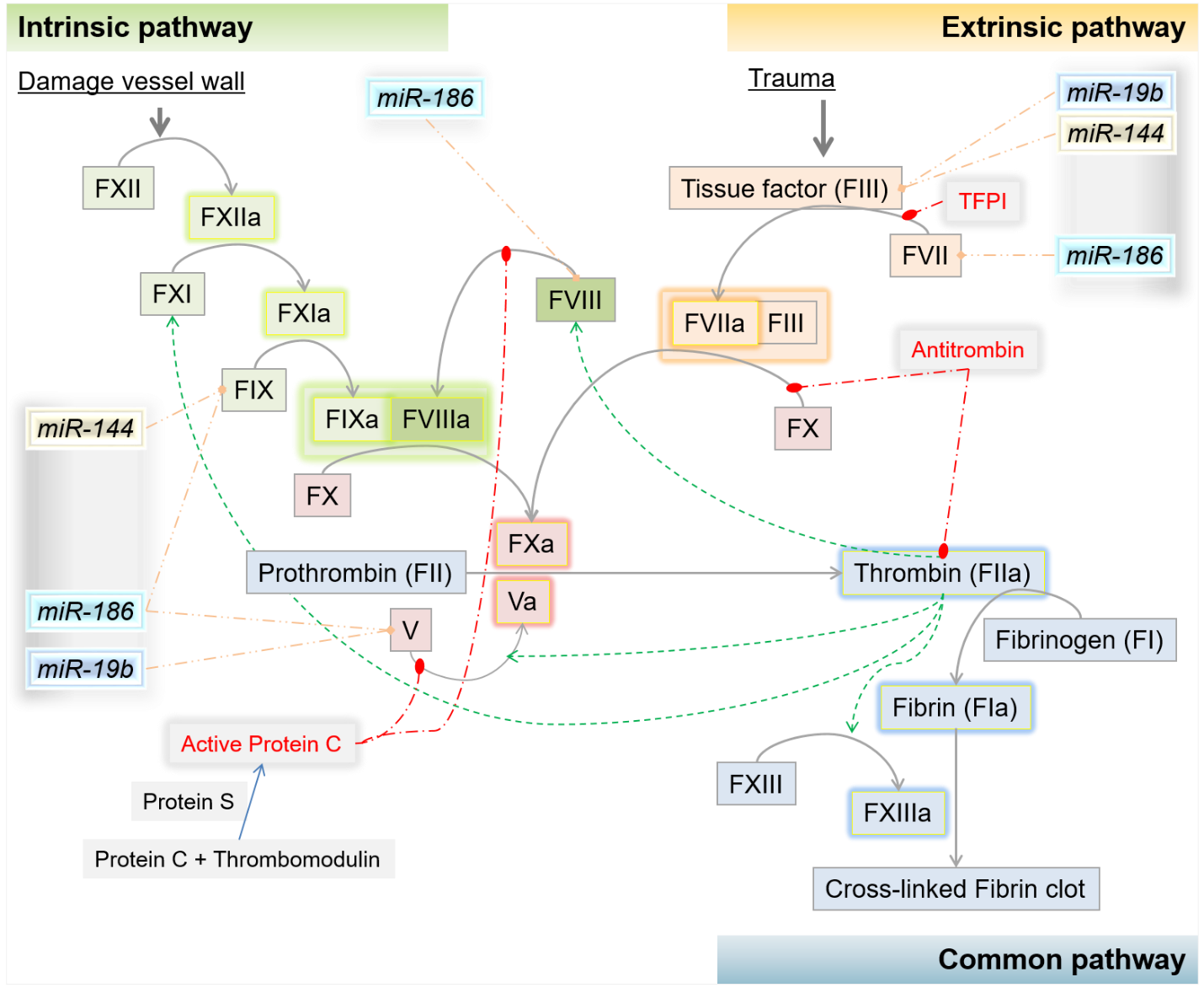
Supplemental Figure S4. Blood coagulation pathway showing the potential protein targets for miR-186, miR-19b-3p and miR-144 as predicted by miRanda (microRNA.org).**
